# Identifying Adverse Events in Outpatients With Prostate Cancer Using Pharmaceutical Care Records in Community Pharmacies: Application of Named Entity Recognition

**DOI:** 10.2196/69663

**Published:** 2025-03-11

**Authors:** Yuki Yanagisawa, Satoshi Watabe, Sakura Yokoyama, Kyoko Sayama, Hayato Kizaki, Masami Tsuchiya, Shungo Imai, Mitsuhiro Someya, Ryoo Taniguchi, Shuntaro Yada, Eiji Aramaki, Satoko Hori

**Affiliations:** 1 Division of Drug Informatics Keio University Faculty of Pharmacy Tokyo Japan; 2 Nakajima Pharmacy Hokkaido Japan; 3 Nara Institute of Science and Technology Nara Japan; 4 Faculty of Library, Information and Media Science University of Tsukuba Tsukuba Japan

**Keywords:** natural language processing, pharmaceutical care records, androgen receptor axis-targeting agents, adverse events, outpatient care

## Abstract

**Background:**

Androgen receptor axis-targeting reagents (ARATs) have become key drugs for patients with castration-resistant prostate cancer (CRPC). ARATs are taken long term in outpatient settings, and effective adverse event (AE) monitoring can help prolong treatment duration for patients with CRPC. Despite the importance of monitoring, few studies have identified which AEs can be captured and assessed in community pharmacies, where pharmacists in Japan dispense medications, provide counseling, and monitor potential AEs for outpatients prescribed ARATs. Therefore, we anticipated that a named entity recognition (NER) system might be used to extract AEs recorded in pharmaceutical care records generated by community pharmacists.

**Objective:**

This study aimed to evaluate whether an NER system can effectively and systematically identify AEs in outpatients undergoing ARAT therapy by reviewing pharmaceutical care records generated by community pharmacists, focusing on assessment notes, which often contain detailed records of AEs. Additionally, the study sought to determine whether outpatient pharmacotherapy monitoring can be enhanced by using NER to systematically collect AEs from pharmaceutical care records.

**Methods:**

We used an NER system based on the widely used Japanese medical term extraction system MedNER-CR-JA, which uses Bidirectional Encoder Representations from Transformers (BERT). To evaluate its performance for pharmaceutical care records by community pharmacists, the NER system was first applied to 1008 assessment notes in records related to anticancer drug prescriptions. Three pharmaceutically proficient researchers compared the results with the annotated notes assigned symptom tags according to annotation guidelines and evaluated the performance of the NER system on the assessment notes in the pharmaceutical care records. The system was then applied to 2193 assessment notes for patients prescribed ARATs.

**Results:**

The *F*_1_-score for exact matches of all symptom tags between the NER system and annotators was 0.72, confirming the NER system has sufficient performance for application to pharmaceutical care records. The NER system automatically assigned 1900 symptom tags for the 2193 assessment notes from patients prescribed ARATs; 623 tags (32.8%) were positive symptom tags (symptoms present), while 1067 tags (56.2%) were negative symptom tags (symptoms absent). Positive symptom tags included ARAT-related AEs such as “pain,” “skin disorders,” “fatigue,” and “gastrointestinal symptoms.” Many other symptoms were classified as serious AEs. Furthermore, differences in symptom tag profiles reflecting pharmacists’ AE monitoring were observed between androgen synthesis inhibition and androgen receptor signaling inhibition.

**Conclusions:**

The NER system successfully extracted AEs from pharmaceutical care records of patients prescribed ARATs, demonstrating its potential to systematically track the presence and absence of AEs in outpatients. Based on the analysis of a large volume of pharmaceutical medical records using the NER system, community pharmacists not only detect potential AEs but also actively monitor the absence of severe AEs, offering valuable insights for the continuous improvement of patient safety management.

## Introduction

According to the International Agency for Research on Cancer, 20 million new cancer cases were reported in 2022 [1]. In particular, an increasing number of patients are receiving chemotherapy at home [2], driven by the increasing availability of oral anticancer drugs over the past 20 years [3]. Outpatient chemotherapy offers advantages such as reduced invasiveness of administration and fewer hospital visits. However, compared with inpatient chemotherapy, the lack of direct and frequent observation by health care professionals can present safety challenges. In practice, ensuring the safety of oral anticancer drugs prescribed to patients is never easy for treating physicians [4,5]. Additionally, for patients, the adverse events (AEs) experienced after the initiation of treatment may be more burdensome than those reported in clinical trials [6]. Therefore, continuous medical support is essential, extending beyond the hospital setting and involving collaboration with community health care services. Community pharmacies, as the most accessible health care providers, play a crucial role in monitoring AEs in outpatients using oral anticancer drugs [7].

Among outpatient chemotherapy options, endocrine therapy is a common treatment for patients with prostate cancer. In particular, for patients with castration-resistant prostate cancer (CRPC), androgen receptor axis-targeting reagents (ARATs)—abiraterone acetate, apalutamide, darolutamide, and enzalutamide—are taken long term in outpatient settings. Therefore, effective AE monitoring can contribute to prolonged treatment duration [8]. Furthermore, ARATs exhibit distinct AE profiles depending on their pharmacological mechanisms [9,10]. Abiraterone acetate, an androgen synthesis inhibitor, has been reported to cause AEs such as hypertension, gastrointestinal symptoms, fatigue, and liver dysfunction [11]. On the other hand, apalutamide, darolutamide, and enzalutamide, which act through androgen receptor (AR) signaling inhibition, have been associated with AEs such as fatigue and dermatologic disorders [12-15].

In Japan, outpatients prescribed ARATs typically have their prescriptions filled at community pharmacies. Pharmacists at these pharmacies dispense medications, provide patient counseling, monitor for potential AEs, and document pharmaceutical care records. Pharmaceutical care records are commonly written in the SOAP (subjective, objective, assessment, and plan) format [16]. In particular, the assessment section contains pharmacists’ evaluations related to the patient's pharmacotherapy and thus may be a fruitful source of information about AEs experienced by patients. By systematically collecting and analyzing the AEs and pharmacists’ assessments accumulated in pharmaceutical care records, it could be possible to clarify the AEs experienced outside the hospital by outpatients prescribed ARATs.

However, pharmaceutical care records comprise huge amounts of unstructured text data, including medical terms, accumulated over time for individual patients, making them difficult to analyze manually. Therefore, natural language processing technology, particularly named entity recognition (NER), offers a solution by enabling the extraction of patients’ illnesses and symptom data from unstructured medical records [17,18]. Although studies using the NER system to analyze medical texts in hospital inpatients have been reported in the past, few studies have focused on outpatient care, especially in community pharmacy settings, where pharmacists contribute to the safe delivery of pharmacotherapy.

This study aimed to determine whether pharmacotherapy monitoring of outpatients prescribed ARATs can be carried out using NER to systematically collect AEs from pharmaceutical care records, with a particular focus on assessment notes, which may contain detailed records of AEs experienced by patients.

## Methods

### Outline

**Figure 1:**
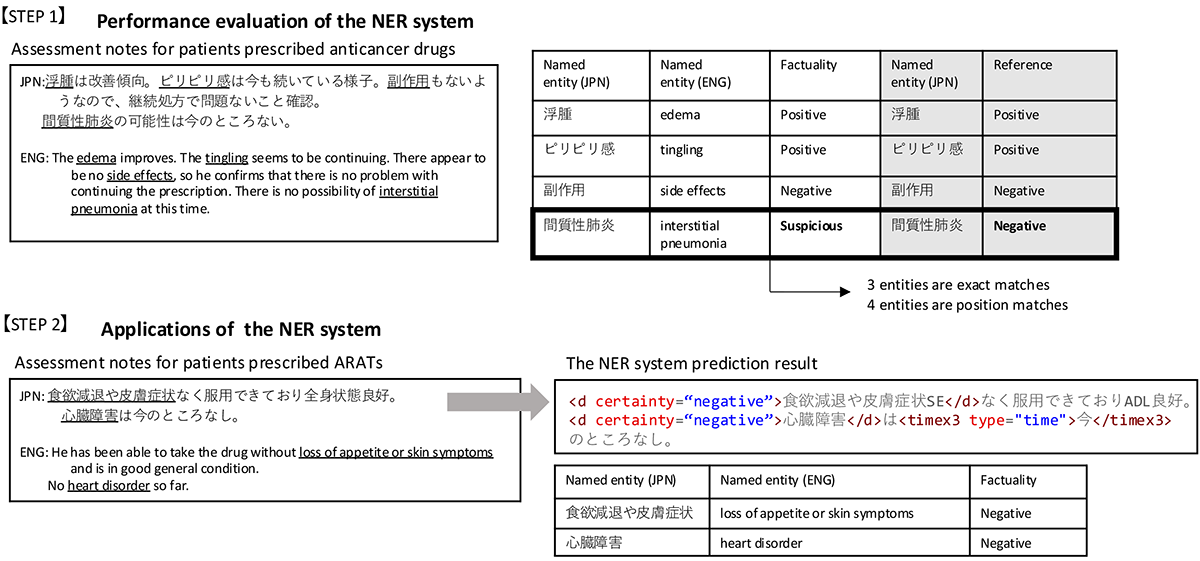
Outline of the experimental method, including manual annotation on a data set, examination of the model's performance in terms of "position match" and "exact match," and use of the named entity recognition (NER) system to extract symptoms from the pharmaceutical care records of patients prescribed androgen receptor axis-targeting agents (ARATs). ENG: English; JPN: Japanese.

An overview of the experimental method is shown in Figure 1. First, we evaluated the performance of the NER system using assessment notes from pharmaceutical care records (STEP 1). The NER system was then used to extract symptoms from the pharmaceutical care records of patients prescribed ARATs (STEP 2).

### Model Description

#### System

Our objective was to evaluate the utility of pharmacist-assessed symptoms extracted from pharmaceutical care records kept by community pharmacies. To extract terms, we applied MedNER-CR-JA, an existing Japanese medical term-extraction system based on Bidirectional Encoder Representations from Transformers (BERT) and that was trained using Japanese case reports [19]. Given the input (pharmaceutical care records), the NER system output the symptoms with the factuality (e.g., positive symptom, negative symptom). The factuality was classified into 4 categories as shown in Table 1.

**Table 1:** Certainty attributes of symptom tags.

Symptom tags	Definition
Positive	The symptom is observed in the patient.
Suspicious	The symptom is suspected to be present in the patient.
Negative	The symptom is not observed in the patient.
General	The symptom is described without reference to the patient's condition.

#### Materials

This study used data from patients at community pharmacies (n=291,150) belonging to the Nakajima Pharmacy Group in Japan from April 2020 to December 2021. The patients’ data consisted of medication orders (structured data) and pharmaceutical care records written in Japanese (unstructured data, n=2,180,902).

First, to evaluate the performance of the NER system (STEP 1), we selected pharmaceutical care records of patients with at least one prescription for anticancer drugs according to the structured data, using the drug codes (YJ codes). YJ codes starting with “42” in Japan indicate anticancer drugs. To evaluate the NER system, we took data recorded during October 2021 through December 2021 at 11 randomly selected pharmacies from the pharmacies with a history of anticancer drug prescriptions. Second, to apply the NER system to pharmaceutical care records (STEP 2), we extracted data for patients prescribed ARATs at least once from April 2020 through December 2021 using individual YJ codes (Multimedia Appendix 1) and used their assessment notes.

In both experiments (STEP 1 and STEP 2), we input the preprocessed text into the NER system. For text preprocessing, we removed line breaks and full-width and half-width spaces from the target text and normalized the text (Unicode). Furthermore, structured sections with template-based input were excluded, and only free-text sections were used.

#### Performance Evaluations and Metrics for Pharmaceutical Care Records

To evaluate the performance of the NER system on the assessment notes in the pharmaceutical care records, we performed manual annotation on a data set. We verified that the NER system was able to assign symptom tags according to existing annotation guidelines [20] with manual annotation performed by 3 pharmaceutically proficient researchers (SY, YY, KS). These researchers were selected based on their pharmaceutical expertise. Two of them were licensed pharmacists with over 5 years of experience in hospitals or pharmacies, while the third was a pharmacy student who had completed a clinical internship in both a hospital and a pharmacy. To ensure consistency and reliability in the study, all researchers were expected to be familiar with the existing guidelines regarding the study objectives, task procedures, and evaluation criteria. The existing guidelines were standardized to enable annotation even by nonmedical professionals. To evaluate the consistency and generalizability of the annotation guidelines across annotators, we randomly extracted 100 cases from the target data, and these were annotated. Label agreement was evaluated using the Fleiss kappa (κ) coefficient to assess the concordance rate among the 3 researchers. Let *Po* be the mean of the raters’ agreement and *Pe* the degree of concentration for each rating, then the calculation formula is as follows:



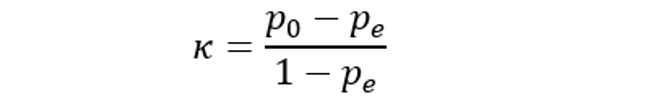



For a difficult task like NER, a score of around 0.6 is considered a substantial match, and the obtained evaluations are considered reasonably reliable [21].

After evaluating their agreement, the 3 researchers (SY, YY, KS) annotated the entire data set. The model’s performance was evaluated using “position match” and “exact match.” Position match is a method that accepts a match with the correct data if the tag's position and name are correct, while exact match requires the tag’s position, name, and attributes to match with the correct data. In the position match evaluation, if the position is correct but the predicted tag name is different from the correct tag name, it is considered incorrect. Therefore, in the exact match, we calculated evaluation metrics separately for tags and attributes, while in the position match, we calculated metrics only for the tags. In both methods, the performance was evaluated in terms of precision, recall, and *F_1_*-score:



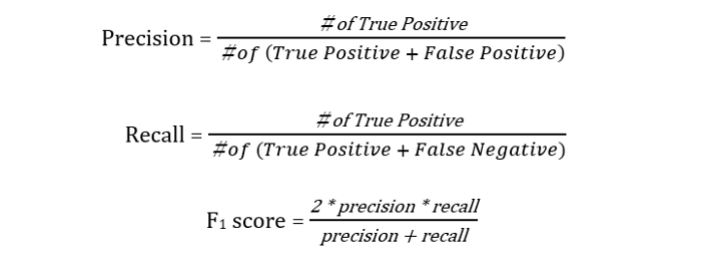



### Ethical Considerations

This study was conducted with anonymized data following approval by the ethics committee of the Keio University Faculty of Pharmacy (approval number: 240618-1) and was conducted in accordance with the relevant guidelines and regulations and the Declaration of Helsinki. Informed consent specific to this study was waived due to the retrospective observational design of the study based on the approval by the ethics committee of the Keio University Faculty of Pharmacy. To respect the will of each stakeholder, however, we provided patients and pharmacists of the pharmacy group with an opportunity to refuse the sharing of their pharmaceutical care records by posting an overview of this study at each pharmacy store or on their websites.

## Results

### Model Performance and Statistics

The data set used to evaluate the performance of the NER system consisted of 1008 assessment notes. The average word count per text in the target data was 48.3 words, with a median of 42.0 words and minimum and maximum values of 4 and 292 words, respectively. The κ coefficient among the 3 annotators for 100 randomly extracted texts was 0.62.

The *F*_1_-score (NER excluding position matches and attribute classification) was 0.72. This score is the macro-average of the *F*_1_-scores for all attribute classifications (positive, suspicious, negative, and general). For positive and negative symptom tags, the *F*_1_-scores were 0.70 and 0.78, respectively (Table 2). The *F*_1_-scores for all attribute classifications are shown in Multimedia Appendix 2. The position matches showed an excellent *F*_1_-score of 0.86 (Multimedia Appendix 2).

**Table 2:** Exact matches between the named entity recognition system and annotators.

Tags	Precision	Recall	*F*_1_*-*score
All symptom tags	0.66	0.78	0.72
Positive symptom tags	0.60	0.85	0.70
Negative symptom tags	0.73	0.83	0.78

### Assessment Notes for Patients Prescribed Antiandrogens

**Figure 2:**
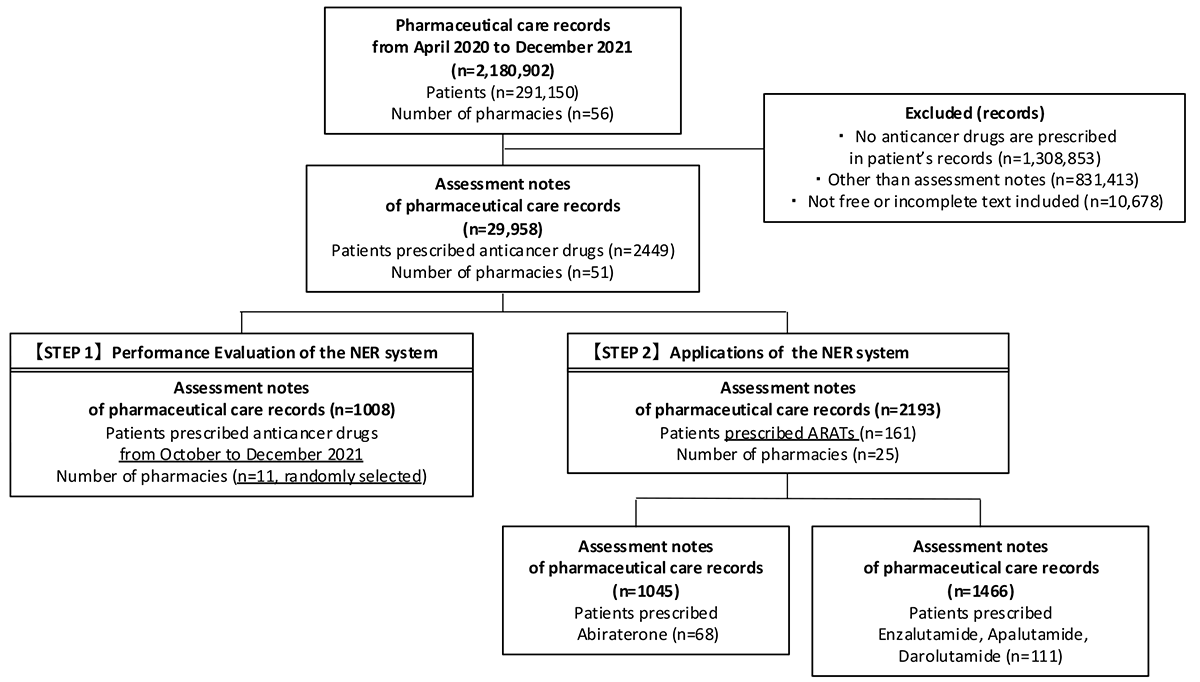
Flowchart for selecting pharmaceutical care records to evaluate the performance of the named entity recognition (NER) system (STEP 1) and apply the NER system to pharmaceutical care records (STEP 2). ARAT: androgen receptor axis-targeting agent.

Figure 2 shows the flowchart of the procedure for selecting pharmaceutical care records. From April 2020 through December 2021, 161 patients had at least one ARAT prescription and corresponding assessment notes in their pharmaceutical care records. There were 2193 assessment notes recording pharmacists’ assessments. Additionally, 68 patients had a history of prescriptions for the androgen synthesis inhibitor abiraterone acetate, with 1045 assessment notes. There were 111 patients with a history of prescription for AR signal transduction inhibitors enzalutamide, apalutamide, and darolutamide, with 1466 assessment notes. On average, each extracted assessment note contained 39.8 words.

### Prediction by the NER System

From 2193 assessment notes of patients prescribed ARATs, the NER system automatically assigned 1900 symptom tags. Among these 1900 symptom tags, 623 (32.8%) were positive symptom tags, predicting the presence of symptoms, while the majority, 1067 (56.2%), were negative symptom tags, predicting the absence of symptoms. Additionally, of the 1900 symptom tags, there were 131 (6.9%) general symptom tags and 79 (4.2%) suspicious symptom tags. Specifically, the positive symptom tags were commonly assigned to notes describing “pain,” “skin disorders,” “fatigue,” and “gastrointestinal symptoms.” The negative symptom tags frequently appeared in descriptions indicating general side effects such as “SE” or “side effects,” accounting for 13.9% (148/1067) of the total. Furthermore, entities with nonpositive symptom tags were AE expressions related to ARATs, such as gastrointestinal symptoms, neuropsychiatric symptoms, and cardiovascular symptoms (Table 3). Other extracted symptoms included seizures and other neuropsychiatric symptoms, cardiovascular symptoms, and hepatic dysfunction. There were also descriptions of severe AEs, including myelosuppression, interstitial pneumonia, and rhabdomyolysis.

Next, the results were examined according to the pharmacological mechanism of action of the ARATs. For patients prescribed the androgen synthesis inhibitor abiraterone acetate, 876 symptom tags were automatically assigned to 1045 assessment notes. The most common symptom tag was negative, with 488 tags (488/876, 55.7%), while 283 positive symptom tags were assigned (283/876, 32.3%; Multimedia Appendix 3). Of the 876 tags, there were 64 (7.3%) general symptom tags and 41 (4.7%) suspicious symptom tags. Regarding characteristic expressions for patients taking abiraterone acetate, “congestive heart failure” was frequently noted among the positive symptom tags. Entities related to skin disorders, which occur frequently in patients taking AR signal transduction inhibitors, appeared infrequently in the top 20 entities. Additionally, entities such as “congestive heart failure,” “drug-induced liver injury,” and “hyperglycemia” were extracted among nonpositive symptom tags.

For patients prescribed the AR signal transduction inhibitors enzalutamide, apalutamide, and darolutamide, 1466 symptom tags were automatically assigned to 1274 assessment notes. Negative symptom tags were most common, amounting to 692 (692/1466, 47.2%), while positive symptom tags amounted to 438 (438/1466, 29.9%; Multimedia Appendix 4). Of the 1466 tags, there were 96 (6.5%) general symptom tags and 48 (3.3%) suspicious symptom tags. Among the positive symptom tags, characteristic entities for AR signal transduction inhibitors such as “skin disorders” and “fatigue” were frequently noted. Additionally, entities such as “seizures,” “psychiatric symptoms,” and “cardiovascular diseases” were extracted among the nonpositive symptom tags.

**Figure 3:**
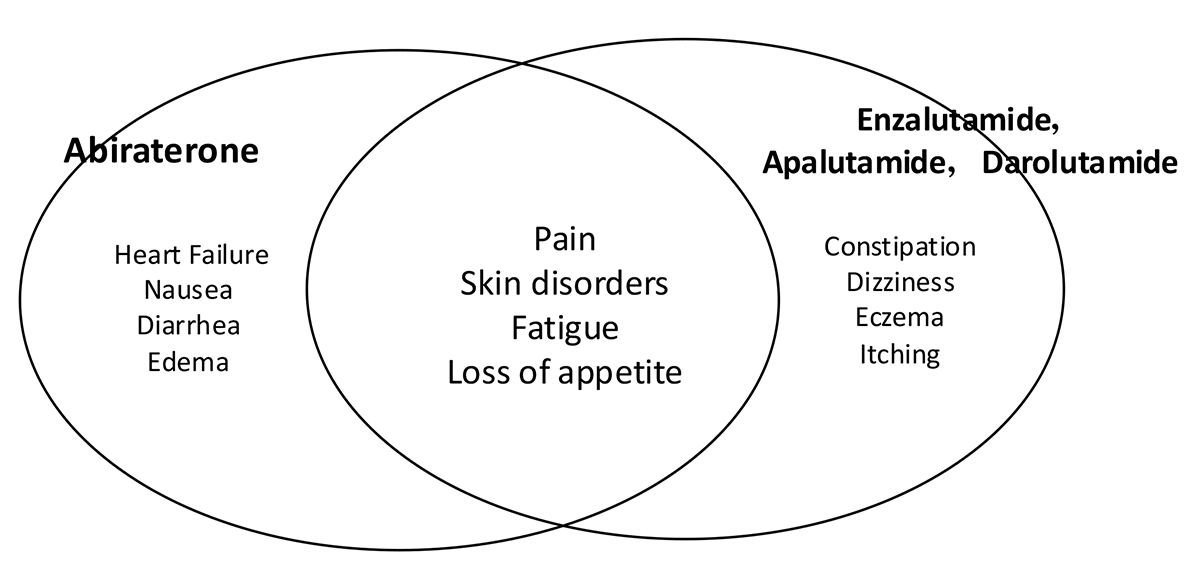
Main symptom entities extracted as positive for agents with different pharmacological mechanisms of action, including androgen synthesis inhibitors (abiraterone acetate) and androgen receptor signaling inhibitors (enzalutamide, apalutamide, darolutamide), shown in order of the number of adverse events extracted.

The differences in symptom entities collected based on the pharmacological mechanism of prescribed ARATs are illustrated in Figure 3.

**Table 3:** Application of the named entity recognition (NER) system to assessment notes of patients prescribed androgen receptor axis-targeting agents (ARATs), showing the top 20 entities.

Entity (Japanese)		Results, n (%)
**Positive symptom tags (n=623)**		
	Pain (痛み)	22 (3.5)
	Skin disorders (皮膚障害)	22 (3.5)
	Fatigue (倦怠感)	16 (2.6)
	Loss of appetite (食欲不振)	15 (2.4)
	Pain (疼痛)	12 (1.9)
	Prostate cancer (前立腺癌)	11 (1.8)
	Diarrhea (下痢)	10 (1.6)
	Poor compliance (コンプライアンス不良)	10 (1.6)
	Gastrointestinal symptoms (消化器症状)	9 (1.4)
	Constipation (便秘)	9 (1.4)
	Liver function disorders (肝機能障害)	8 (1.3)
	Itching (痒み)	8 (1.3)
	Congestive heart failure (うっ血性心不全)	8 (1.3)
	Hypoglycemia (低血糖)	7 (1.1)
	Dizziness (ふらつき)	7 (1.1)
	Skin symptoms (皮膚症状)	7 (1.1)
	Decreased appetite (食欲低下)	6 (1)
	Dry mouth (口渇)	6 (1)
	Decreased PSA^a^ (PSA低下)	6 (1)
	Nausea (嘔気)	6 (1)
**Negative symptom tags (n=1067)**		
	SE (SE)^b^	117 (11)
	Gastrointestinal symptoms (消化器症状)	90 (8.4)
	Neuropsychiatric symptoms (精神神経症状)	32 (3)
	Side effects (副作用)	31 (2.9)
	Changes in physical condition (体調変化)	27 (2.5)
	Cardiovascular symptoms (循環器症状)	22 (2.1)
	Good adherence (アドヒア良好)	21 (2)
	Adherence (アドヒア)	17 (1.6)
	Unpleasant symptoms (不快な症状)	17 (1.6)
	Symptom changes (症状変化)	16 (1.5)
	Missed dose (飲み忘れ)	14 (1.3)
	Side effect symptoms (副作用症状)	13 (1.2)
	Compliance (コンプライアンス)	13 (1.2)
	Adverse events (有害事象)	12 (1.1)
	Side effect symptoms (SE症状)	12 (1.1)
	Progress in physical condition (体調問題)	11 (1)
	Bleeding tendency (出血傾向)	11 (1)
	Hypoglycemia (低血糖)	11 (1)
	Pain (疼痛)	11 (1)
	Elevated PSA (PSA上昇)	11 (1)

^a^PSA: prostate-specific antigen.

^b^SE (SE): Side effects.

## Discussion

### Overview

We evaluated the performance of the NER system on pharmaceutical care records and successfully extracted symptoms, including AEs, from the free-text assessment notes of community pharmacists. This study is the first to report the use of the NER system to systematically identify AEs from pharmaceutical care records of patients prescribed ARATs.

### Predictive Performance of the NER System

The NER system exhibited robust performance on the pharmaceutical care records analyzed. Although the kappa statistics for annotation (0.62) indicate substantial agreement, the following factors were considered to have contributed to the decrease in the kappa. The most common type of disagreement among annotators was related to the selection range of the entity. Specifically, the annotation guidelines [16] we referenced set the standard for selecting the entire range when symptoms were expressed in parallel. However, discrepancies in the interpretation of the range occurred among the annotators. For example, for text such as “gastrointestinal symptoms, hypoglycemia present,” disagreements arose over whether to annotate this as “gastrointestinal symptoms” and “hypoglycemia” or as “gastrointestinal symptoms, hypoglycemia.”

The *F*_1_-score for symptom tagging (NER and attribute classification) was 0.72, and the *F*_1_-scores for positive and negative predictive performance ranged from 0.70 to 0.78. These results indicate that the system is effective for analyzing the pharmaceutical care records used in this study. Notably, the system outperformed prior work by Ohno et al [22], who reported an *F*_1_-score of 0.64 for assessment notes among the SOAP format descriptions in Japanese hospital-based pharmaceutical care records using MedNER-J (NER and positive and negative classification). The NER evaluation method used in this study assumed that entities that completely matched the annotations were correct. In addition, many types of tags were used, and the evaluation was conducted under more stringent conditions.

Several factors may have affected model performance. In particular, there were specific expressions in pharmaceutical care records that we recognized as causing frequent errors in NER predictions during the performance evaluation. First, pharmacist assessment records often include descriptions related to medication adherence. The NER system sometimes incorrectly recognized terms such as “adherence” and “compliance” as symptom entities (for example, the entity “adherence” was predicted as a symptom expression). Second, pharmacist assessment records frequently included descriptions related to AE confirmation, often using nuanced expressions characteristic of Japanese, such as “no suspicion of XX.” As a result, the NER system occasionally predicted “suspicious” for negative symptom expressions (for example, the entity “interstitial pneumonia” was predicted as suspected in the sentence “no suspicion of interstitial pneumonia,” whereas the correct status should be negative). Additionally, the system showed low extraction rates for entities related to hypertension, which is a side effect specific to enzalutamide acetate, as well as blood glucose–related expressions associated with concomitant corticosteroids and expressions related to hypokalemia, possibly because vital sign–related terms like “blood pressure” and “blood glucose” were treated as test values rather than symptoms. Another challenge was interpreting negation patterns, such as “No symptoms: XX (Symptoms)” and “Symptoms (-),” which may have led to incorrect symptom tagging.

### AEs Identified in Pharmaceutical Care Records for Patients Prescribed ARATs

Our analysis of pharmacists’ assessment records for outpatients prescribed ARATs revealed that over 90% of the records contained symptom tags. The extracted entities predominantly indicated symptoms related to AEs commonly seen in patients treated with ARATs. Common positive symptom entities included “pain,” “skin disorders,” “fatigue,” “anorexia,” and other typical side effects of ARATs. “Pain” was the most frequently identified symptom, reflecting the high prevalence of bone metastases in patients with CRPC requiring ARAT therapy. A study using the Frankfurt Metastatic Cancer Database of the Prostate reported that 78% of patients with CRPC had bone metastases [23].

On the other hand, the fact that the top 20 entities accounted for less than one-third of the total positive symptom tags suggests that many symptoms occur with low frequency, resulting in a long-tail distribution. This indicates that a large portion of symptom tags is dispersed across numerous less-frequent entities. This distribution indicates that the NER system has the sensitivity to capture a wide range of symptoms. However, it also suggests the need for standardization if the aim is to extract specific symptom expressions.

Entities other than positive symptom tags were serious AEs such as “interstitial pneumonia,” “myelosuppression,” and “rhabdomyolysis.” Interestingly, many of the negative symptom tags indicated the absence of severe AEs, suggesting that pharmacists were actively monitoring for signs of AEs and documenting the absence of signs. These negative assessments, particularly for rare but serious AEs, highlight the thoroughness of pharmacists in ensuring the safety of outpatient pharmacotherapy.

### Drug-Specific Monitoring by Community Pharmacists

There are two types of ARATs with different pharmacological mechanisms of action: androgen synthesis inhibition and AR signaling inhibition. Each category is associated with distinct AEs, which require tailored care. Despite the common indication for ARATs, this study highlights the differences in pharmacists’ monitoring based on drug type.

The most characteristic positive symptom extracted for abiraterone acetate, an androgen synthesis inhibitor, was “heart failure,” a critical AE requiring close monitoring. In addition, “cardiovascular symptoms,” “congestive heart failure,” “rhabdomyolysis,” and “drug-induced liver injury” were also identified as nonpositive symptoms.

For patients prescribed AR signaling inhibitors (enzalutamide, apalutamide, darolutamide), the most frequently extracted positive symptoms included “skin problems,” “fatigue,” and “gastrointestinal symptoms,” which are common AEs that are important to monitor. “Skin disorder” was more frequently reported as a symptom for AR signaling inhibitors, whereas it was less commonly reported for abiraterone acetate, an androgen synthesis inhibitor. In addition, “gastrointestinal symptoms” and “neuropsychiatric symptoms” were extracted from the nonpositive symptom tags. “Seizures” were also extracted from the nonpositive symptom tags. Seizures are a serious AE specifically associated with AR signaling inhibitors [15,24,25].

These results indicate that, even though ARATs are used for the same purpose, pharmacists should pay particular attention to certain AEs depending on the pharmacological mechanism of each type of ARAT.

### Comparison With Prior Work

The NER system used in this study has previously been reported to be applicable to medical records documented in electronic medical records within hospitals in Japan [22,26,27]. For example, Ohno et al [22] reported an *F*_1_-score of 0.64 for an NER system applied to hospital-based pharmaceutical records. In contrast, this study applied the NER system to pharmaceutical records documented in community pharmacies outside hospitals and achieved an *F*_1_-score of 0.72 under more stringent evaluation conditions.

A key feature of this study is its focus on pharmaceutical care records by community pharmacists, which tend to include more diverse and nuanced expressions of outpatients than electronic medical records. Furthermore, our previous studies have demonstrated that patients with hand-foot syndrome and serious AEs can be identified using NLP applied to pharmaceutical care records by community pharmacies [28]. In this study, the NER system enabled the extraction of a wide range of AEs, not limited to specific side effects. These results highlight advancements in NER methodologies and underscore their applicability in real-world outpatient settings.

### Limitations

This study has several limitations. First, the content of pharmaceutical care records may vary depending on the pharmacists' experience, the role of each pharmacy, and the condition of the patients. Second, this study focused on the extraction of AEs and did not include symptom normalization. As a result, synonymous entities such as “pain” were counted separately. Third, the pharmacies included in this study were part of a single pharmacy group, which may limit the generalizability of the findings to other pharmacy settings.

### Prospects

The NER system used in this study enabled the automated analysis of a large volume of records accumulated by community pharmacists. This system can extract symptoms from text, determine their factuality, and monitor patients’ symptoms over time. Additionally, error analysis of the NER system applied to pharmaceutical medical records by community pharmacists can contribute to the appropriate and effective use of NER systems in the future.

Our findings support the idea that NER technology offers the potential for real-time and longitudinal monitoring of patients’ symptoms through the seamless integration of local health care systems, including community pharmacies. In hospital electronic health records, it has been demonstrated that the accuracy of detecting AEs improves when physicians’ notes are combined with records from nurses and pharmacists [23]. Similarly, for outpatients, AEs identified by community pharmacists may play a key role in effective monitoring. This approach would extend beyond the confines of hospital-based electronic medical record systems. By using NER technology, it should be possible to gain detailed insights into drug efficacy and the progression of AEs. Moreover, it should enable earlier interventions and timely adjustments to treatment plans, particularly for outpatients who may otherwise have limited direct interaction with health care providers

### Conclusions

We evaluated the performance of the NER system on Japanese medical text, focusing on pharmaceutical care records by community pharmacists. The NER system successfully extracted AEs from pharmaceutical care records. This is the first study to apply NER to the pharmaceutical care records of patients prescribed ARATs in community settings. Our analysis of pharmacists’ assessment records for outpatients prescribed ARATs revealed that over 90% of the records contained symptom tags. Notably, ARATs have distinct pharmacological mechanisms and exhibit different AE profiles, and the NER system successfully captured these variations. Furthermore, it highlights the role of community pharmacists in monitoring specific AEs related to ARAT therapy and, importantly, in documenting the absence of severe AEs as well. This study demonstrates that NER can effectively capture symptoms reported by outpatients and documented by community pharmacists, complementing hospital records and contributing to a more comprehensive understanding of AEs in outpatient settings. The NER system is expected to be a valuable tool for enhancing pharmacotherapy monitoring in outpatient settings.

## Appendix

Multimedia Appendix 1 Codes for Targeted Pharmaceuticals.

Multimedia Appendix 2 Exact and partial matches between the NER system and the annotators.

Multimedia Appendix 3 Application of the NER system to assessment notes of patients prescribed abiraterone acetate.

Multimedia Appendix 4 Application of the NER system to assessment notes of patients prescribed enzalutamide, apalutamide, darolutamide.

## Abbreviations

AE: adverse event
AR: androgen receptor
ARAT: androgen receptor axis-targeting agent
BERT: Bidirectional Encoder Representations from Transformers
CRPC: castration-resistant prostate cancer
NER: named entity recognition
SOAP: subjective, objective, assessment, and plan

## Data Availability

All data generated or analyzed during this study are included in this published article.
