# Supplementary material for: Identifying Adverse Events in Outpatients With Prostate Cancer Using Pharmaceutical Care Records in Community Pharmacies: Application of Named Entity Recognition
Source: JMIR Cancer. 2025 Mar 11;11:e69663. doi: 10.2196/69663 (PMC11937706; doi:10.2196/69663)
Supplement: Multimedia Appendix 1 [file cancer_v11i1e69663_app1.pdf]

Multimedia Appendix 1

Codes for Targeted Pharmaceuticals

Drug Name	Generic Name	ATC* code	YJ code
Zytiga® Tablets 250mg	Abiraterone Acetate	L02BX03	4291033F1024
Zytiga® Tablets 500mg	Abiraterone Acetate	L02BX03	4291033F2020
Erleada® Tablets 60mg	Apalutamide	L02BB05	4291059F1029
Xtandi® Tablets 40mg	Enzalutamide	L02BB04	4291031F1025
Xtandi® Tablets 80mg	Enzalutamide	L02BB04	4291031F2021
Nubeqa® Tablets 300mg	Darolutamide	L02BB06	4291063F1025

* ATC: Anatomical Therapeutic Chemical Classification
