# Supplementary material for: Identifying Adverse Events in Outpatients With Prostate Cancer Using Pharmaceutical Care Records in Community Pharmacies: Application of Named Entity Recognition
Source: JMIR Cancer. 2025 Mar 11;11:e69663. doi: 10.2196/69663 (PMC11937706; doi:10.2196/69663)
Supplement: Multimedia Appendix 2 [file cancer_v11i1e69663_app2.pdf]

Multimedia Appendix 2

Exact matches between the NER system and the annotators

	Precision	Recall	*F1*-score
All symptom tags	0.66	0.78	0.72
Positive symptom tags	0.60	0.85	0.70
Suspicious symptom tags	0.19	0.24	0.21
Negative symptom tags	0.73	0.83	0.78
General symptom tags	0.50	0.39	0.44

Partial matches between MedNER-J and the annotators

	Precision	Recall	*F1*-score
All symptom tags	0.79	0.94	0.86
