# Supplementary material for: Identifying Adverse Events in Outpatients With Prostate Cancer Using Pharmaceutical Care Records in Community Pharmacies: Application of Named Entity Recognition
Source: JMIR Cancer. 2025 Mar 11;11:e69663. doi: 10.2196/69663 (PMC11937706; doi:10.2196/69663)
Supplement: Multimedia Appendix 3 [file cancer_v11i1e69663_app3.pdf]

Multimedia Appendix 3

Application of the NER system to assessment notes of patients prescribed abiraterone acetate (Symptom tags, n = 876)

Positive symptom tags, n = 283		Negative symptom tags, n = 488	
The top 20 Entities, (JPN)	n (%)	The top 20 Entities, (JPN)	n (%)
Pain (痛み)	12 (4.2)	SE (SE)	43 (8.8)
Prostate cancer (前立腺癌)	8 (2.8)	Gastrointestinal symptoms (消化器症状)	23 (4.7)
Pain (疼痛)	8 (2.8)	Side Effect (副作用)	17 (3.5)
Congestive heart failure (うっ血性心不全)	8 (2.8)	Cardiovascular symptoms (循環器症状)	16 (3.3)
Nausea (嘔気)	6 (2.1)	Changes in physical condition (体調変化)	10 (2.0)
Liver function disorders (肝機能障害)	6 (2.1)	Changes in symptoms (症状変化)	10 (2.0)
Loss of appetite (食欲不振)	5 (1.8)	Pain (疼痛)	10 (2.0)
Diarrhea (下痢)	5 (1.8)	Subjective symptoms suspected to be SE (SE と思われるような自覚症状)	10 (2.0)
Hypokalemia (低カリウム血症)	3 (1.1)	Complaints of SE (SE の訴え)	9 (1.8)
Drug-resistant hypersensitivity (薬剤耐性過敏症)	3 (1.1)	Neuropsychiatric symptoms (精神神経症状)	8 (1.6)
Bone metastasis (骨転移)	3 (1.1)	Unpleasant symptoms (不快な症状)	8 (1.6)
Constipation (便秘)	3 (1.1)	Compliance (コンプライアンス)	8 (1.6)
Fever (発熱)	3 (1.1)	Good adherence (アドヒア良好)	8 (1.6)
Poor compliance (コンプライアンス不良)	3 (1.1)	Side effect symptoms (副作用症状)	7 (1.4)
Fatigue (倦怠感)	3 (1.1)	SE symptoms (SE 症状)	7 (1.4)
Getting nauseated (むかつき)	3 (1.1)	Adherence (アドヒア)	6 (1.2)
Dizziness (ふらつき)	3 (1.1)	Adverse events (有害事象)	6 (1.2)
Eczema (湿疹)	3 (1.1)	Drowsiness (眠気)	6 (1.2)
Skin disorders (皮膚障害)	3 (1.1)	Continuation (継続)	5 (1.0)
Edema (浮腫み)	3 (1.1)	electrolyte disturbance (電解質異常)	4 (0.8)
