# Supplementary material for: Identifying Adverse Events in Outpatients With Prostate Cancer Using Pharmaceutical Care Records in Community Pharmacies: Application of Named Entity Recognition
Source: JMIR Cancer. 2025 Mar 11;11:e69663. doi: 10.2196/69663 (PMC11937706; doi:10.2196/69663)
Supplement: Multimedia Appendix 4 [file cancer_v11i1e69663_app4.pdf]

Multimedia Appendix 4

Application of the NER system to assessment notes of patients prescribed enzalutamide, apalutamide, darolutamide (Symptom tags, n = 1,466)

Positive symptom tags, n = 438		Negative symptom tags, n = 692	
The top 20 Entities, (JPN)	n (%)	The top 20 Entities, (JPN)	n (%)
Skin disorders (皮膚障害)	22 (5.0)	Side effect (SE)	86 (12.4)
Fatigue (倦怠感)	13 (3.0)	Gastrointestinal symptoms (消化器症状)	81 (11.7)
Pain (痛み)	12 (2.7)	Psychiatric and neurological symptoms (精神神経症状)	29 (4.2)
Pain (疼痛)	11 (2.5)	Changes in physical condition (体調変化)	17 (2.5)
Loss of appetite (食欲不振)	10 (2.3)	Changes in symptoms (症状変化)	16 (2.3)
Gastrointestinal symptoms (消化器症状)	9 (2.1)	Good adherence (アドヒア良好)	15 (2.2)
Constipation (便秘)	8 (1.8)	Side Effect (副作用)	15 (2.2)
Prostate cancer (前立腺癌)	7 (1.6)	Adherence (アドヒア)	11 (1.6)
Poor compliance (コンプライアンス不良)	7 (1.6)	Missed dose (飲み忘れ)	11 (1.6)
Itching (痒み)	7 (1.6)	Cardiovascular symptoms (循環器症状)	11 (1.6)
Hypoglycemia (低血糖)	6 (1.4)	Unpleasant symptoms (不快な症状)	10 (1.4)
Dizziness (ふらつき)	6 (1.4)	Progress in physical condition (体調問題)	10 (1.4)
Eczema (湿疹)	6 (1.4)	Bleeding tendency (出血傾向)	10 (1.4)
Elevated blood pressure (血圧上昇)	6 (1.4)	Interactions (相互作用)	9 (1.3)
Skin symptoms (皮膚症状)	6 (1.4)	Hypoglycemia (低血糖)	9 (1.3)
Diarrhea (下痢)	5 (1.1)	Side effect symptoms (副作用症状)	9 (1.3)
Decreased PSA (PSA 低下)	5 (1.1)	Increased PSA (PSA 上昇)	9 (1.3)
Dry mouth (口渇)	5 (1.1)	Cardiac dysfunction (心機能障害)	7 (1.0)
Adherence (アドヒア)	4 (0.9)	Drug (薬剤)	7 (1.0)
Fractures (骨折)	4 (0.9)	Side effect occurrence (副作用発現)	7 (1.0)
